# Exploring insertions and deletions (indels) of *MSRB3* gene and their association with growth traits in four Chinese indigenous cattle breeds

**DOI:** 10.5194/aab-62-465-2019

**Published:** 2019-07-25

**Authors:** Mingli Wu, Shipeng Li, Guoliang Zhang, Yingzhi Fan, Yuan Gao, Yongzhen Huang, Xianyong Lan, Chuzhao Lei, Yun Ma, Ruihua Dang

**Affiliations:** 1 Key Laboratory of Animal Genetics, Breeding and Reproduction of Shaanxi Province, College of Animal Science and Technology, Northwest A&F University, Yangling, Shaanxi, 712100, P. R. China; 2 Branch of Animal Husbandry, Jilin Academy of Agricultural Science, Gongzhuling, Jilin, 136100, P. R. China; 3 College of Life Sciences, Xinyang Normal University, Xinyang, Henan, 464000, P. R. China; 4 School of Agriculture, Ningxia University, Yinchuan, Ningxia, 750021, P. R. China

## Abstract

Methionine sulfoxide reductase B3 (*MSRB3*) is instrumental in ossification and fat deposition, which regulate the growth and development of cattle directly. The purpose of this study was aimed to explore insertions and deletions (indels) in *MSRB3* gene and investigate their association with growth traits in four indigenous cattle breeds (Luxi cattle, Qinchuan cattle, Nanyang cattle, and Jiaxian Red cattle). Four indels were identified by sequencing with DNA pool. Association analysis showed that three of them were associated with growth traits (P<0.05). For P1, the DD (deletion and deletion) genotype was significantly associated with body length of Nanyang cattle; for P6, II (insertion and insertion) and/or DD genotypes were significantly associated with enhanced growth traits of Qinchuan cattle; for P7, II genotype was significantly associated with hucklebone width of Luxi cattle. Our results demonstrated that the polymorphisms in bovine *MSRB3* gene were significantly associated with growth traits, which could be candidate loci for marker-assisted selection (MAS) in cattle breeding.

## 1 Introduction

There are abundant indigenous cattle breeds in China and most of them were draft cattle for thousands of years (Zhang et al., 2018). Chinese indigenous cattle breeds have the characteristics of slow growth, low yield, and low-grade beef (Yue et al., 2019). With the rapid development of livestock husbandry, beef cattle improvement has become a heated and difficult problem (Singh et al., 2011). However, because of the slow progress in traditional breeding, we need to seek a more efficient and accurate way of breeding urgently (Gui et al., 2015). A noteworthy method is molecular breeding technology, which emerged and developed into an indispensable breeding method, and indels is one of the genetic markers used for assisted selection (Zhang et al., 2003; Fan et al., 2007).

The identification of candidate genes for growth traits has attracted more and more attention. *MSRB3* (methionine sulfoxide reductase B3) is an essential member of the *MSRB* gene family, which can reduce the catalysis effect of methionine-R-sulfoxide to methionine specifically as an oxidoreductase (Kwak et al., 2017a). Previous research has shown that *MSRB3* gene could influence ear shape and size, otitis media, heat resistance, oxidation resistance, auditory system, reproductive traits, and size of the hippocampus (Zhang et al., 2017; Fan et al., 2007; Pillas et al., 2010; Shen et al., 2015; Wei et al., 2015; Lee et al., 2012). In recent years, based on the analysis of GWASs (genome-wide association studies), gene silencing, and protein precipitation, *MSRB3* was regarded as a candidate gene to affect bovine growth traits (Saatchi et al., 2014). At the same time, it inhibited the proliferation of mouse embryonic fibroblasts and human dermal fibroblasts by acting on the p53, p21, and p27 pathways (Lee et al., 2014; Kwak et al., 2017b).

Consequently, the purpose of this research is to obtain mutation information on *MSRB3* gene and its association with the body size of Chinese indigenous cattle through the indel screening and association analysis with growth traits.

## 2 Materials and methods

All experiments performed in this study were approved by the International Animal Care and Use Committee of the Northwest A&F University (IACUC-NWAFU). Furthermore, the care and use of animals was fully compliant with local animal welfare laws, guidelines, and policies.

### 2.1 DNA samples and data collection

A total of 673 Chinese indigenous cattle were used in this study, including Luxi cattle (LX, N=113, Shandong Province), Qinchuan cattle (QC, N=286, Shaanxi Province), Nanyang cattle (NY, N=135, Henan Province), and Jiaxian Red cattle (JX, N=139, Henan Province) (Huang et al., 2017, 2018). All individuals selected were between 2 and 5 years old, healthy, and unrelated. Blood samples were collected in anticoagulant blood vessel and stored at -80 ${}^{\circ }$C (Zhao et al., 2017a). In order to demonstrate the relationship between polymorphisms and performance traits, more than 6000 growth traits were measured and calculated, including body weight (BW), body height (BH), body length (BL), chest circumference (ChC), chest depth (ChD), chest width (ChW), hucklebone width (HuW), hip width (HW), cannon circumference (CaC), body length index (BLI), chest circumference index (ChCI), chest width index (ChWI), cannon circumference index (CaCI), hucklebone width index (HuWI), and trunk index (TI) (Zhang et al., 2019; Zhao et al., 2018). All data were measured by the same person following the same standard (Zhao et al., 2018).

### 2.2 DNA isolation and genomic DNA pools construction

Genomic DNA was isolated from leukocytes and the method refers to a whole-blood genomic DNA extraction kit (Aidlab Biotechnologies Co., Ltd., China). DNA quantity and purity (OD${}_{260}$ / OD${}_{280}$) for each sample was assessed by a NanoDrop^™^ 1000 spectrometer (Thermo Scientific, Waltham, MA, USA). DNA samples were diluted to a standard concentration (50 ng µL${}^{-1}$) and stored at -80 ${}^{\circ }$C. To explore the allele variation of the bovine *MSRB3* gene, every 25 samples were mixed and used for polymerase chain reaction (PCR) (Zhao et al., 2017b).

### 2.3 Indel loci detection and genotyping

Based on the whole-genome sequencing of Chinese indigenous cattle in our lab, seven possible indel loci were found in cattle *MSRB3* gene and their PCR primers were designed by Primer Premier 5.0 (Premier Biosoft International, USA) (Table 1) (Chen et al., 2018). PCR amplification was performed in a 20 µL volume system: 1 µL DNA (50 ng µL${}^{-1}$), 10 µL 2× PCR mix (Beijing ComWin Biotech Co., Ltd., China), 0.5 µM of each primers, and 8 µL ${\mathrm{ddH}}_{2}O$. The amplification conditions of PCR were listed as follows: denaturing at 95 ${}^{\circ }$C for 5 min followed by 35 cycles of 95 ${}^{\circ }$C for 30 s, annealing for 30 s, and extending at 72 ${}^{\circ }$C for 30 s followed by final extension at 72 ${}^{\circ }$C for 10 min. PCR products were sequenced directly (Shanghai Sangon Biotech Co., Ltd., P. R. China) to identify potential mutation sites (Zhao et al., 2018). For the confirmed indel loci, every sample was amplified by the aforementioned PCR system and genotyped by 10 % PAGE (polyacrylamide gel electrophoresis) (Pu et al., 2014).

**Table 1:** Primers designed for detecting indels of *MSRB3* gene in cattle.

Loci	Primer sequences (5${}^{\prime }$–3${}^{\prime }$)	Tm (${}^{\circ }$C)	Product size	Notes
P1	F: ACGCACTGTATGATTCCA	54	161 bp	Pool DNA sequencing
	R: ATAGGCCAAGATAGAGGC			or indel genotyping
P2	F: ATACAGGAAAGACAAAGAGGGG	50	284 bp	Pool DNA sequencing
	R: GAGAATGGATGCTGGATGATAG			or indel genotyping
P3	F: GGCATATTACTCACTCAACA	50	402 bp	Pool DNA sequencing
	R: CACAAACCAGCTTAGACTTT			
P4	F: GATCCCTGACTGGAGAACTAG	52	292 bp	Pool DNA sequencing
	R: CACTGTGACCCAGACCCT			
P5	F: GTGAAGCCCTAAATGAGA	50	223 bp	Pool DNA sequencing
	R: GTTGTATGGAATGGGAGTA			
P6	F: GCATAAGAAAGCCAACCT	54	221 bp	Pool DNA sequencing
	R: CAGCCTCATCATCTCATCCA			or indel genotyping
P7	F: GAGCCCTAATGGATAAAA	51	332 bp	Pool DNA sequencing/
	R: AGTGTTGAAGTTGCCTGT			indel genotyping

Notes: indel, insertions and deletions; *MSRB3,* methionine sulfoxide reductase B3; F, forward primer; R, reverse primer; Tm, melting temperature; P1–P7, first pair to seventh pair.

### 2.4 Statistical analyses

The sequences were contrasted and analyzed by Bioedit software (UK). Hardy–Weinberg equilibrium (HWE), homozygosity (Ho), heterozygosity (He), effective allele numbers (Ne), and polymorphism information content (PIC) were calculated and analyzed using the MSRcall website (http://www.msrcall.com/, last access: 25 August 2018), which was based on Nei's method (Koehl et al., 2018; Yeh et al., 1999; Botstein et al., 1980; Nei, 1973). Distribution differences for genotypic and allelic frequencies among different breeds were analyzed using the $\chi^{2}$ test implemented in SPSS (version 18.0; IBM Corp., Armonk, NY, USA). Linkage disequilibrium was performed by the SHEsis online platform (http://analysis.bio-x.cn, last access: 25 August 2018) (Li et al., 2009). SPSS software (version 18.0) (International Business Machines, US) was used to calculate the associations between different genotypes and growth traits; the structure of the model is $Y_{ijk}=\mu +G_{i}+B_{j}+e_{ijk}$, where $Y_{ijk}$ is the phenotypic observations, μ is the mean of the phenotypic observations, $G_{i}$ is the effect of genotype of the jth observation, $B_{j}$ is the effect of breed in the ith observation, and $e_{ijk}$ is the residual effect (Liu et al., 2016; Shi et al., 2016).

## 3 Results

### 3.1 Variant screening and genotyping of bovine *MSRB3* gene

**Figure 1:**
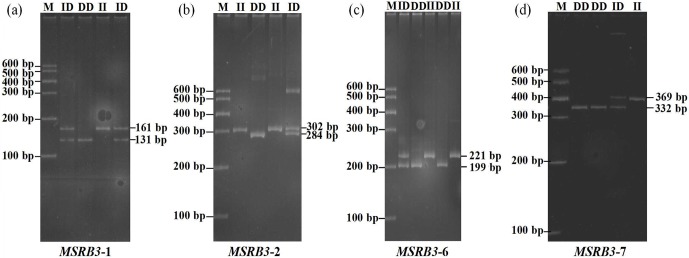
The agarose gel electrophoresis patterns of four indel loci in cattle *MSRB3* gene.

**Figure 2:**
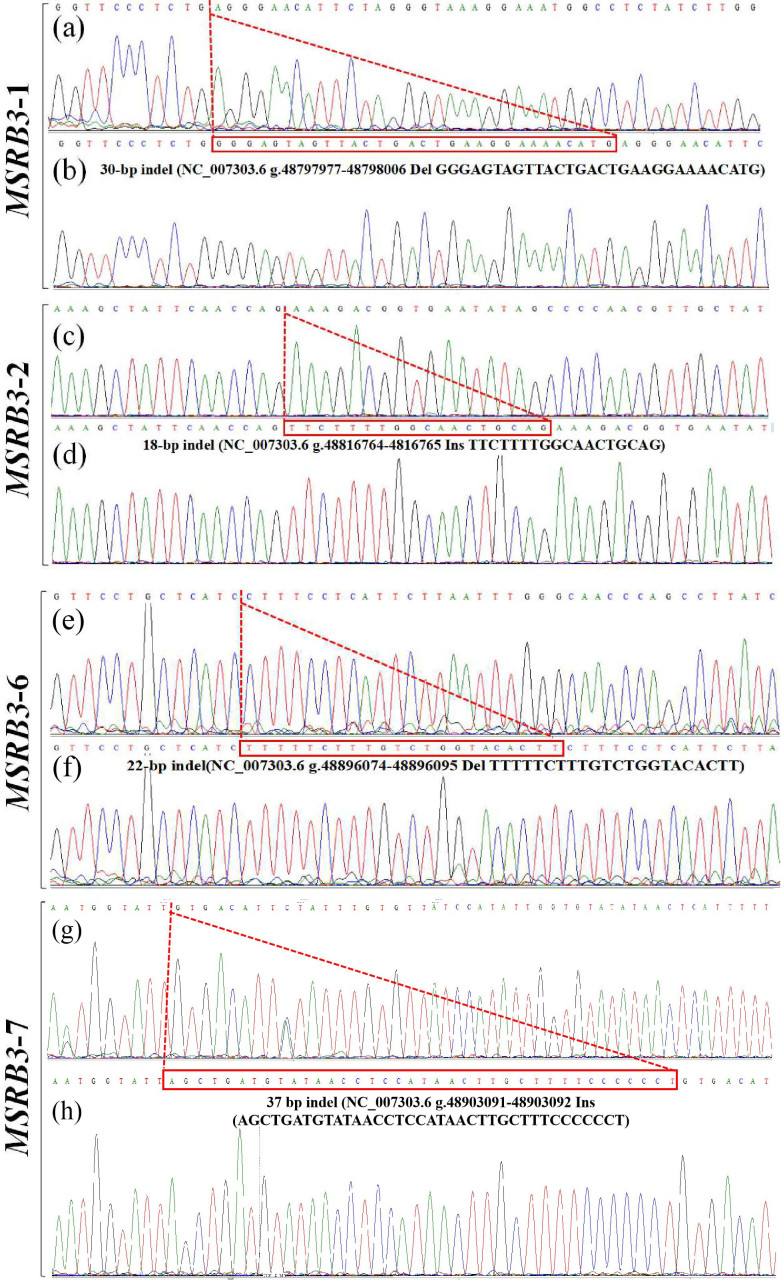
Sequencing maps of four indel loci in cattle *MSRB3* gene. **(a)** Homozygotic deletion and deletion type (ID) of *MSRB3*-1 locus; **(b)** homozygotic insertion and insertion type (II) of *MSRB3*-1 locus, the sequence with a dashed red line boundary is a 30 bp deletion; **(c)** homozygotic deletion and deletion type (ID) of *MSRB3*-2 locus; **(d)** homozygotic insertion and insertion type (II) of *MSRB3*-2 locus, the sequence with a dashed red line boundary is a 18 bp insertion; **(e)** homozygotic deletion and deletion type (ID) of *MSRB3*-6 locus; **(f)** homozygotic insertion and insertion type (II) of *MSRB3*-6 locus, the sequence with a dashed red line boundary is a 22 bp deletion; **(g)** homozygotic deletion and deletion type (ID) of *MSRB3*-7 locus; **(h)** homozygotic insertion and insertion type (II) of *MSRB3*-7 locus, the sequence with a dashed red line boundary is a 37 bp insertion.

In our previous study, four indel loci (P1, P2, P6, and P7) were identified from seven potential variations in bovine *MSRB3* gene. Respectively, P1 was a 30 bp deletion (NC_007303.6 g.48797977-48798006 del GGGAGTAGTTACTGACTGAAGGAAAACATG); P2 was a 18 bp insertion (NC_007303.6 g.48816764-48816765 ins TTCTTTTGGCAACTGCAG); P6 was a 22 bp deletion (NC_007303.6 g.48896074-48896095 del TTTTTCTTTGTCTGGTACACTT); P7 was a 37 bp insertion (NC_007303.6 g.48903091-48903092 ins AGCTGATGTATAACCTCCATAACTTGCTTTCCCCCCT); and no variation was found in P3, P4, or P5 (Fig. 1, 2). All the samples were genotyped by PCR-AFLP (amplified fragment length polymorphism). As shown in the Fig. 1a, wild type (II) showed a 161 bp band, mutant (DD) showed a 131 bp band, and heterozygote (ID) showed 131 and 161 bp bands; as shown in the Fig. 1b, wild type (DD) showed a 284 bp band, mutant (II) showed a 302 bp band, and heterozygote (ID) showed 284 and 302 bp bands; as shown in the Fig. 1c, wild type (II) showed a 221 bp band, mutant (DD) showed a 199 bp band, and heterozygote (ID) showed 199 and 221 bp bands; as shown in the Fig. 1d, wild type (DD) showed a 332 bp band, mutant (II) showed a 369 bp band, and heterozygote (ID) showed 369 and 332 bp bands.

**Table 2:** Genotypes, allele and population genetic information of four indel loci in *MSRB3* gene.

Loci	Breeds	Sizes	Genotypic			Allelic		HWE	Population parameters			
			frequencies			frequencies						
		N	II	ID	DD	I	D	P values	Ho	He	Ne	PIC
P1	LX	107	59	44	4	0.757	0.243	P>0.05	0.632	0.368	1.582	0.300
	QC	284	240	43	1	0.921	0.079	P>0.05	0.854	0.146	1.171	0.135
	NY	128	85	38	5	0.812	0.188	P>0.05	0.854	0.146	1.171	0.135
	JX	139	107	29	3	0.874	0.126	P>0.05	0.780	0.220	1.282	0.196
P2	LX	112	5	52	55	0.277	0.723	P>0.05	0.600	0.400	1.668	0.320
	QC	286	2	38	246	0.073	0.927	P>0.05	0.864	0.136	1.158	0.127
	NY	130	2	53	75	0.219	0.781	P<0.05	0.658	0.342	1.521	0.284
	JX	137	2	33	102	0.135	0.865	P>0.05	0.766	0.234	1.305	0.206
P6	LX	110	69	41	0	0.814	0.186	P<0.05	0.697	0.303	1.435	0.257
	QC	273	142	90	41	0.685	0.315	P<0.05	0.568	0.432	1.759	0.338
	NY	113	66	41	6	0.765	0.235	P>0.05	0.641	0.359	1.560	0.295
	JX	138	106	29	3	0.873	0.127	P>0.05	0.779	0.221	1.284	0.197
P7	LX	113	4	72	37	0.354	0.646	P<0.05	0.543	0.457	1.843	0.353
	QC	274	3	27	244	0.060	0.940	P<0.05	0.887	0.113	1.128	0.107
	NY	135	6	41	88	0.196	0.804	P>0.05	0.684	0.316	1.461	0.266
	JX	138	0	72	66	0.261	0.739	P<0.05	0.614	0.386	1.628	0.311

Notes: indel, insertions and deletions; *MSRB3,* methionine sulfoxide reductase B3; N, number; HWE, Hardy–Weinberg equilibrium; Ho, homozygosity; He, heterozygosity; Ne, effective allele numbers; PIC, polymorphism information content; LX, Luxi cattle; QC, Qinchuan cattle; NY, Nanyang cattle; JX, Jiaxian Red cattle; II, insertion and insertion; ID, insertion and deletion; DD, deletion and deletion.

**Table 3:** The $\chi^{2}$ test of different breeds on four indels of cattle *MSRB3* gene.

Loci	Types	Breeds	LX	QC	NY	JX
P1		LX	–	$\chi^{2}=39.31$	$\chi^{2}=3.40$	$\chi^{2}=13.16$
	Genotypic	QC	(p<0.01)	–	$\chi^{2}=20.81$	$\chi^{2}=5.66$
	frequencies	NY	(p>0.05)	(p<0.01)	–	$\chi^{2}=3.78$
		JX	(p<0.01)	(p<0.05)	(p>0.05)	–
		LX	–	$\chi^{2}=33.85$	$\chi^{2}=8.95$	$\chi^{2}=45.49$
	Allelic	QC	(p<0.01)	–	$\chi^{2}=51.35$	$\chi^{2}=12.01$
	frequencies	NY	(p<0.01)	(p<0.01)	–	$\chi^{2}=14.52$
		JX	(p<0.01)	(p<0.01)	(p<0.01)	–
P2		LX	–	$\chi^{2}=60.07$	$\chi^{2}=3.05$	$\chi^{2}=17.27$
	Genotypic	QC	(p<0.01)		$\chi^{2}=40.80$	$\chi^{2}=8.51$
	frequencies	NY	(p>0.05)	(p<0.01)	–	$\chi^{2}=8.59$
		JX	(p<0.01)	(p<0.01)	(p<0.01)	–
		LX	–	$\chi^{2}=144.12$	$\chi^{2}=91.38$	$\chi^{2}=61.64$
	Allelic	QC	(p<0.01)	–	$\chi^{2}=389.71$	$\chi^{2}=20.63$
	frequencies	NY	(p<0.01)	(p<0.01)	–	$\chi^{2}=257.34$
		JX	(p<0.01)	(p<0.01)	(p<0.01)	–
P6		LX	–	$\chi^{2}=18.58$	$\chi^{2}=6.03$	$\chi^{2}=9.84$
	Genotypic	QC	(p<0.01)	–	$\chi^{2}=7.05$	$\chi^{2}=27.99$
	frequencies	NY	(p<0.05)	(p<0.01)	–	$\chi^{2}=9.97$
		JX	(p<0.01)	(p<0.01)	(p<0.01)	–
		LX	–	$\chi^{2}=44.32$	$\chi^{2}=7.22$	$\chi^{2}=13.18$
	Allelic	QC	(p<0.01)	–	$\chi^{2}=16.05$	$\chi^{2}=102.65$
	frequencies	NY	(p<0.01)	(p<0.01)	–	$\chi^{2}=39.34$
		JX	(p<0.01)	(p<0.01)	(p<0.01)	–
P7		LX	–	$\chi^{2}=128.31$	$\chi^{2}=27.98$	$\chi^{2}=9.77$
	Genotypic	QC	(p<0.01)	–	$\chi^{2}=33.85$	$\chi^{2}=90.64$
	frequencies	NY	(p<0.01)	(p<0.01)	–	$\chi^{2}=9.97$
		JX	(p<0.01)	(p<0.01)	(p<0.01)	–
		LX	–	$\chi^{2}=263.28$	$\chi^{2}=62.61$	$\chi^{2}=20.31$
	Allelic	QC	(p<0.01)	–	$\chi^{2}=82.86$	$\chi^{2}=149.92$
	frequencies	NY	(p<0.01)	(p<0.01)	–	$\chi^{2}=11.98$
		JX	(p<0.01)	(p<0.01)	(p<0.01)	–

Notes: indel, insertions and deletions; *MSRB3*, methionine sulfoxide reductase B3; LX, Luxi cattle; QC, Qinchuan cattle; NY, Nanyang cattle; JX, Jiaxian Red cattle.

**Table 4:** Haplotypes and their frequencies of four indels in four Chinese cattle breeds.

Haplotype	Hap. seq.	LX number	QC number	NY number	JX number
		(frequency)	(frequency)	(frequency)	(frequency)
Hap 1	P1${}^{I}$ P2${}^{I}$ P6${}^{I}$ P7${}^{I}$	13.76 (0.066)${}^{*}$	–	4.66 (0.019)	4.56 (0.017)
Hap 2	P1${}^{I}$ P2${}^{I}$ P6${}^{I}$ P7${}^{D}$	8.89 (0.043)	8.46 (0.017)	9.82 (0.041)	1.23 (0.005)
Hap 3	P1${}^{I}$ P2${}^{I}$ P6${}^{D}$ P7${}^{I}$	0.13 (0.001)	–	0.39 (0.002)	2.38 (0.009)
Hap 4	P1${}^{I}$ P2${}^{D}$ P6${}^{I}$ P7${}^{I}$	23.37 (0.112)${}^{*}$	3.61 (0.007)	4.27 (0.018)	26.84 (0.100)${}^{*}$
Hap 5	P1${}^{I}$ P2${}^{D}$ P6${}^{I}$ P7${}^{D}$	73.25 (0.352)${}^{*}$	302.82 (0.594)${}^{*}$	129.72 (0.541)${}^{*}$	67.72 (0.626)${}^{*}$
Hap 6	P1${}^{I}$ P2${}^{D}$ P6${}^{D}$ P7${}^{I}$	4.97 (0.024)	1.45 (0.003)	–	7.84 (0.029)
Hap 7	P1${}^{I}$ P2${}^{I}$ P6${}^{D}$ P7${}^{D}$	–	4.85 (0.010)	3.31 (0.014)	–
Hap 8	P1${}^{I}$ P2${}^{D}$ P6${}^{D}$ P7${}^{D}$	33.63 (0.162)${}^{*}$	149.81 (0.294)${}^{*}$	1.99 (0.004)	23.43 (0.087)${}^{*}$
Hap 9	P1${}^{D}$ P2${}^{I}$ P6${}^{I}$ P7${}^{I}$	24.76 (0.119)${}^{*}$	19.08 (0.037)${}^{*}$	27.03 (0.113)${}^{*}$	23.18 (0.086${}^{*}$
Hap 10	P1${}^{D}$ P2${}^{I}$ P6${}^{I}$ P7${}^{D}$	9.46 (0.045)	0.01 (0.000)	4.43 (0.018)	2.84 (0.011)
Hap 11	P1${}^{D}$ P2${}^{I}$ P6${}^{D}$ P7${}^{I}$	–	3.87 (0.008)	–	0.05 (0.000)
Hap 12	P1${}^{D}$ P2${}^{I}$ P6${}^{D}$ P7${}^{D}$	–	0.73 (0.001)	0.35 (0.001)	0.75 (0.003)
Hap 13	P1${}^{D}$ P2${}^{D}$ P6${}^{I}$ P7${}^{I}$	6.76 (0.033)	1.99 (0.004)	5.65 (0.024)	1.61 (0.006)
Hap 14	P1${}^{D}$ P2${}^{D}$ P6${}^{I}$ P7${}^{D}$	8.75 (0.042)	10.02 (0.020)	5.41 (0.023)	5.02 (0.019)
Hap 15	P1${}^{D}$ P2${}^{D}$ P6${}^{D}$ P7${}^{I}$	0.24 (0.001)	–	–	0.54 (0.002)
Hap 16	P1${}^{D}$ P2${}^{D}$ P6${}^{D}$ P7${}^{D}$	0.03 (0.000)	3.29 (0.006)	1.12 (0.005)	–

Notes: indel, insertions and deletions; hap, haplotype; hap. seq., haplotype sequence; LX, Luxi cattle; QC, Qinchuan cattle; NY, Nanyang cattle; JX, Jiaxian Red cattle; I, insertion; D, deletion; ${}^{*}$ means p>0.03.

**Table 5:** Three novel indels in *MSRB3* gene and their associations with growth traits.

Loci	Breeds	Growth traits	Observed genotypes (LSM${}^{a}\pm \mathrm{SE}$)			
			II	ID	DD	P values
P1	NY	Body length (cm)	$139.11\pm 10.36^{\mathrm{ab}}$	$138.13\pm 12.09^{b}$	$152.20\pm 13.23^{a}$	0.05
			(n=85)	(n=38)	(n=5)	
		Chest circumference (cm)	$192.21\pm 1.36^{a}$	$183.08\pm 2.52^{b}$	$189.54\pm 6.13^{\mathrm{ab}}$	0.00
			(n=87)	(n=52)	(n=12)	
		Hip height (cm)	$49.81\pm 0.47^{a}$	$46.82\pm 0.67^{b}$	$46.50\pm 4.67^{b}$	0.01
			(n=77)	(n=50)	(n=11)	
P6	QC	Body weight (kg)	$519.50\pm 9.31^{a}$	$466.80\pm 14.43^{b}$	$504.11\pm 43.90^{\mathrm{ab}}$	0.01
			(n=87)	(n=52)	(n=12)	
		Chest length index	$127.69\pm 0.90^{\mathrm{ab}}$	$122.63\pm 1.97^{b}$	$127.83\pm 2.51^{a}$	0.03
			(n=86)	(n=53)	(n=12)	
		Chest circumference index	$148.70\pm 0.82^{a}$	$144.16\pm 1.63^{b}$	$147.41\pm 3.12^{\mathrm{ab}}$	0.03
			(n=87)	(n=52)	(n=12)	
P7	LX	Hucklebone width (cm)	$30.50\pm 2.90^{a}$	$29.38\pm 0.37^{b}$	$27.80\pm 0.42^{b}$	0.03
			(n=4)	(n=72)	(n=37)	

Notes: indel, insertions and deletions; *MSRB3,* methionine sulfoxide reductase B3; LSM, least squares technique, SE, standard error; LX, Luxi cattle; QC, Qinchuan cattle; NY, Nanyang cattle; II, insertion and insertion; ID, insertion and deletion; DD, deletion and deletion; ${}^{a,\;b}$ means P<0.05.

**Figure 3:**
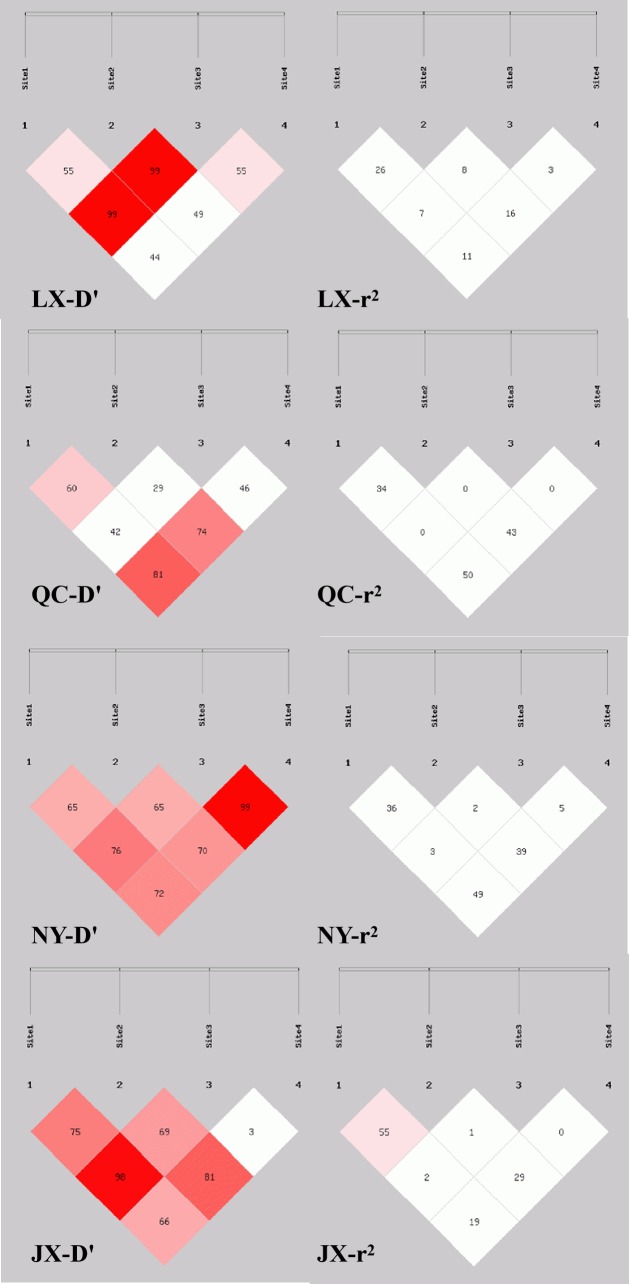
Linkage disequilibrium plot of four indel loci in cattle *MSRB3* gene.

PCR products showed three genotypes in all of the indel loci in the cattle *MSRB3* gene which were detected by 10 % PAGE gel electrophoresis, including (a) the insertion and insertion type (II genotype) consisting of a 161 bp band, deletion and deletion type (DD genotype) consisting of a 131 bp band, and the heterozygote type (ID genotype) that showed 161 and 131 bp bands in *MSRB3*-1 locus; (b) the insertion and insertion type (II genotype) consisting of a 302 bp band, deletion and deletion type (DD genotype) consisting of a 284 bp band, and the heterozygote type (ID genotype) that showed 302 and 284 bp bands in *MSRB3*-2 locus; (c) the insertion and insertion type (II genotype) consisting of a 221 bp band, deletion and deletion type (DD genotype) consisting of a 199 bp band, and the heterozygote type (ID genotype) showed 221 and 199 bp bands in *MSRB3*-6 locus; and (d) the insertion and insertion type (II genotype) consisting of a 369 bp band, deletion and deletion types (DD genotype) consisting of a 332 bp band, and the heterozygote type (ID genotype) that showed 369 and 332 bp bands in *MSRB3*-7 locus.

### 3.2 Population genetic variability analysis in four cattle breeds

Genetic diversity is the prerequisite for species survival, adaptation, and evolution, and is also important for improving the performance of meat production. Genotype frequency, allele frequency, and genetic parameters were estimated and shown in Table 2. For P1 locus, the breeds of QC, NY, and JX belonged to a low polymorphic locus (0<PIC<0.25) and LX belonged to a moderate polymorphic locus (0.25<PIC<0.50). All of them were in Hardy–Weinberg equilibrium (HWE) (P>0.05). Homozygosity (Ho) ranged from 0.632 to 0.854. Heterozygosity (He) ranged from 0.146 to 0.368 and effective allele numbers (Ne) were between 1.171 and 1.582. The I allele (0.757–0.921) was higher than D allele (0.079–0.243) in all four breeds. For P2 locus, LX and NY were intermediately polymorphic because they possessed moderate genetic diversity (0.25<PIC<0.50) and QC and JX belonged to low polymorphic locus (0<PIC<0.25). All of them were in Hardy–Weinberg equilibrium (HWE) except NY (P>0.05). Homozygosity (Ho) ranged from 0.600 to 0.864. Heterozygosity (He) ranged from 0.136 to 0.400 and effective allele numbers (Ne) were between 1.158 and 1.668. The I allele (0.073–0.277) was lower than D allele (0.723–0.927) in all four breeds. For P6 locus, LX and QC population deviated from the Hardy-Weinberg equilibrium among the four populations. The populations of LX, QC, and JX belonged to moderate genetic diversity (0.25<PIC<0.50) but the JX population belonged to low polymorphic diversity (0<PIC<0.25). Homozygosity (Ho) ranged from 0.568 to 0.779. Heterozygosity (He) ranged from 0.221 to 0.432 and effective allele numbers (Ne) were between 1.284 and 1.759. The I allele (0.685–0.873) was higher than D allele (0.127–0.315) in all four breeds. For P7, LX, QC, and JX, populations deviated from the Hardy-Weinberg equilibrium, except the NY population. The populations of LX, NY, and JX belonged to moderate genetic diversity (0.25<PIC<0.50) but the QC population belonged to low polymorphic diversity (0<PIC<0.25). Homozygosity (Ho) ranged from 0.543 to 0.887. Heterozygosity (He) ranged from 0.113 to 0.457 and effective allele numbers (Ne) were between 1.128 and 1.843. The I allele (0.060–0.354) was lower than D allele (0.646–0.940) in all four breeds. The $\chi^{2}$ test showed that there was no significant difference in the genotypic frequencies between LX and NY (P1 locus), NY and JX (P1 locus), or LX and NY (P2, P6 loci) (Table 3). In P1 locus, between QC with JX, and in P6 locus, NY with LX populations, the genotypic frequencies were significantly different, and others were extremely and significantly different. Allele frequencies were significantly different among different breeds in four loci (Table 3).

### 3.3 Haplotype analysis and linkage disequilibrium

Sixteen haplotypes were analyzed in all four breeds. The frequencies are listed in Table 4, only the frequency of “P1${}^{I}$ P2${}^{D}$ P6${}^{I}$ P7${}^{D}$” and “P1${}^{I}$ P2${}^{D}$ P6${}^{D}$ P7${}^{D}$” haplotypes were all 3 % higher than others in four cattle breeds. The results of linkage disequilibrium of four loci in all four breeds are shown in Fig. 3. Between the P1 and P2, P1 and P4, and P2 and P4 pairs, there is a strong linkage in both QC and NY. In the JX population, between P1 and P2, there is also a strong linkage.

### 3.4 Association analysis between indels in *MSRB3* gene and growth traits

Association analysis, shown in Table 5, revealed that loci of P1, P6, and P7 had a significant effect on growth traits. The chest circumference of DD genotype at P1 locus was significantly longer than ID genotype in the NY population (P<0.05). As for P6 locus, significant differences were found in chest circumference, hip height, and body weight in QC cattle (P<0.01); the II genotype or DD genotype of body length index and chest circumference index showed better performance in the QC population (p<0.05). As for P6 locus, II genotype benefited hucklebone width of the LX population. The linkage disequilibrium showed that P1 and P7 were demonstrated a strong linkage in QC cattle and NY cattle ($r^{2}>0.33$) (Fig. 3).

## 4 Discussion

In previous studies, the function of *MSRB3* is related to ear morphology, auditory system, cell proliferation, and apoptosis (Kwak et al., 2017b; Lee et al., 2014). The results of GWASs have shown that *MSRB3* is involved in regulation of ossification and adipose tissue development in cattle (Saatchi et al., 2014). Some studies have shown that the knockout of *MSRB3* leads to a decrease in fibroblast proliferation, which could influence the function of proteins, glycosaminoglycan and glycoproteins (Lee et al., 2014). Further study revealed that inhibition of *MSRB3* could activate the expression of *P53*, and enhance the expression level of *P21* and *P27* subsequently (Kwak et al., 2017b). Above-mentioned studies strongly suggest that *MSRB3* inhibited cell proliferation by acting on p53, p21, and p27 pathways, which suggests that *MSRB3* is an important candidate gene for animal growth and development (Kwak et al., 2017b; Lee et al., 2014; Eujin et al., 2014). However, there was no study on marker-assisted selection in bovine *MSRB3* gene. Therefore, we further verified the relationship between four indel loci in bovine *MSRB3* gene and their growth traits.

In our study, P6 was published by NCBI (National Center of Biotechnology Information) and the remaining loci were newly discovered. The $\chi^{2}$ test showed that the P1 was in Hardy-Weinberg equilibrium but P2, P6, and P7 were not in different cattle breeds. The reason may be that artificial selection was carried out to obtain better classification characters, the genetic background of different varieties was inconsistent, or smaller sample size (Doekes et al., 2018; Zhou et al., 2018; Capellini et al., 2017). Through genetic diversity estimation, the classification of polymorphism information content (PIC) resulted in the fact that four loci were in a low polymorphism class in most of the analyzed breeds, the others were identified as having moderate polymorphism. This means that most local cattle have not been bred at these sites. On the basis of two popular measures ($r^{2}$ and D${}^{\prime }$) performed in the linkage disequilibrium analysis, all pairs of the P1, P2, and P7 in QC and NY populations demonstrated a strong linkage. Also, a strong linkage between P1 and P2 in the JX population was shown, and others showed a weak linkage. Association analysis shows that three were related to growth traits and there is no evidence that P2 is related to the growth of cattle. P1 caused a 30 bp deletion so as to affect body length in LX. Body length is an important index of meat production; therefore, it can be proved that P1 can be used as a novel marker for breeding (Lacerda et al., 2018; Xu et al., 2018). The deletion of P6 led to a decrease in multiple growth traits of the QC population. Growth traits of II genotype were better than ID and DD genotypes. Both P1 and P6 had harmful mutations. Association analysis of P7 resulted in the fact that individual cattle with II genotype had superior growth traits compared to individuals with ID and DD genotypes. The indels of *MSRB3* included functional and nonfunctional types. Functional indels affect the gene product and growth traits, whereas nonfunctional indels may be strongly linked with functional indels of *MSRB3*.

In a nutshell, the indels of P1, P6, and P7 within *MSRB3* gene significantly affected growth traits, meaning that P1, P6, and P7 could be used as DNA markers for eliminating or selecting excellent individuals in marker-assisted selection (MAS) breeding in relation to growth traits, and *MSRB3* gene could be used as a candidate gene for breeding beef cattle.

## 5 Conclusions

This research identified four indels within *MSRB3* gene firstly, which were associated with notable growth traits in four Chinese indigenous cattle breeds. Meanwhile, functional indels could be utilized as promising molecular markers in early marker-assisted selection for cattle breeding after the further extensive research.

## Data Availability

Data are available upon request.
